# Supplementary material for: Determination of Optimal Predictors and Sampling Frequency to Develop Nutrient Soft Sensors Using Random Forest
Source: Sensors (Basel). 2023 Jun 30;23(13):6057. doi: 10.3390/s23136057 (PMC10346477; doi:10.3390/s23136057)
Supplement: Supplementary file 1 [file sensors-23-06057-s001.zip › sensors-2414080-supplementary.pdf]

Supporting Information for

Determination of optimal predictors and sampling frequency to develop nutrient soft sensors using random forest

Muhammad Arhab, Jingshui Huang *

Chair of Hydrology and River Basin Management, Technical University of Munich, Arcisstrasse 21, 80333 Munich, Germany

* Correspondence: jingshui.huang@tum.de

SI contains two tables and two figures.

Table S1. Pearson coefficient between dependent variables and predictors for Erlabrunn and Kahl am Main stations.

Station: Kahl am Main						Station: Erlabrunn			
NO_3-N		OPO_4-P		NH_4-N		NO_3-N		OPO_4-P	
month_sin	0.83	pH	-0.66	week_cos	0.30	month_sin	0.81	month_sin	-0.31
Temp	-0.77	O2	-0.66	month_cos	0.29	Temp	-0.79	week_sin	-0.30
week_sin	0.73	month_sin	-0.61	Temp	-0.27	O2	0.75	O2	-0.29
O2	0.68	week_sin	-0.55	O2	0.20	week_sin	0.68	Temp	0.19
week_cos	0.53	Temp	0.51	Flow	0.09	week_cos	0.60	Flow	0.16
Flow	0.44	week_cos	-0.32	Conduct	0.04	month_cos	0.41	Conduct	-0.11
Conduct	-0.38	Flow	-0.23	month_sin	0.04	Conduct	-0.15	week_cos	-0.09
month_cos	0.33	month_cos	-0.17	week_sin	-0.03	Flow	0.11	pH	-0.05
pH	0.11	Conduct	0.11	pH	0.01	pH	0.06	month_cos	-0.01

Table S2. Hyperparameters tuning results

Parameter	Kahl am Main			Erlabrunn	
	NO_3-N	OPO_4-P	NH_4	NO_3-N	OPO_4-P
Bootstrap	True	True	True	True	True
Depth of the trees	30	20	30	30	30
Minimum number of samples to split a node	12	6	6	12	6
Minimum number of samples to be at a leaf node	20	6	6	6	6

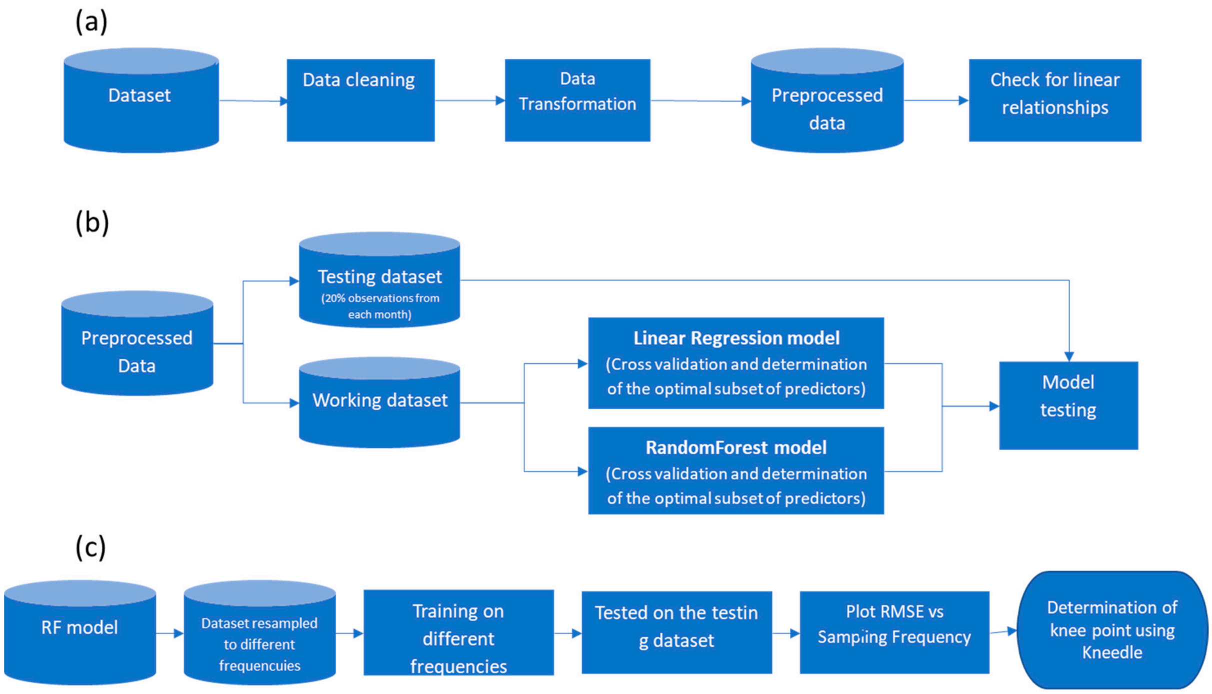

Figure S1. Flowchart illustrating the study's progression: (a) Data cleaning procedure, (b) Model development, and (c) Identification of the optimal sampling frequency.

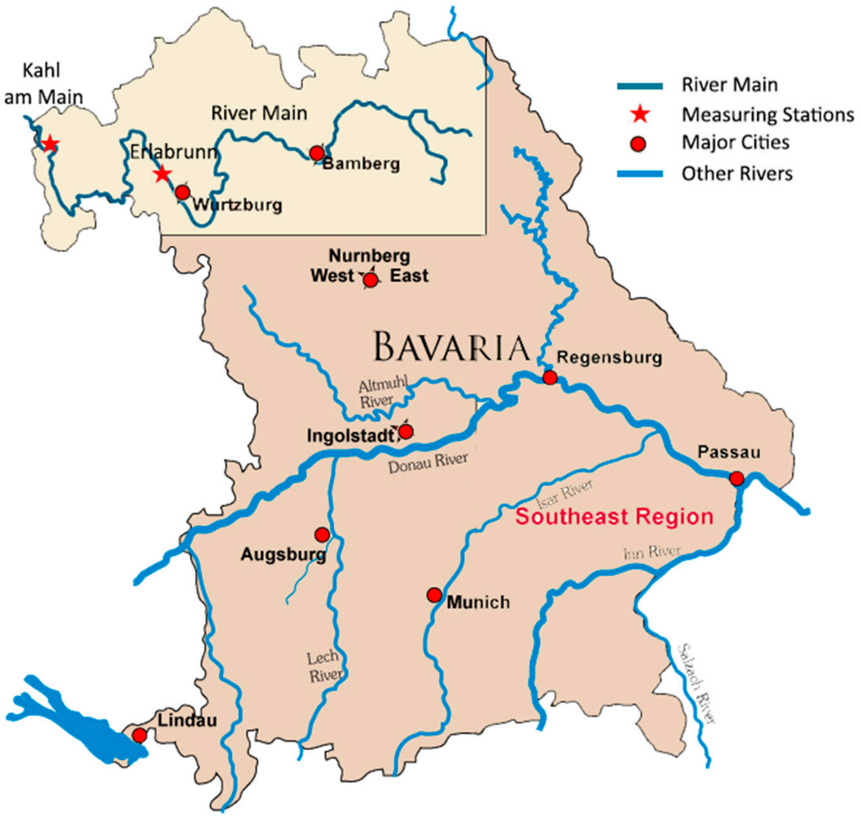

Figure S2. Map of Bavaria with the study site highlighted on the top left
